# Supplementary material for: Characterization of Date Seed Powder Derived Porous Graphene Oxide and Its Application as an Environmental Functional Material to Remove Dye from Aqueous Solutions
Source: Materials (Basel). 2022 Nov 16;15(22):8136. doi: 10.3390/ma15228136 (PMC9693346; doi:10.3390/ma15228136)
Supplement: Supplementary file 1 [file materials-15-08136-s001.zip › materials-1990728-supplementary.pdf]

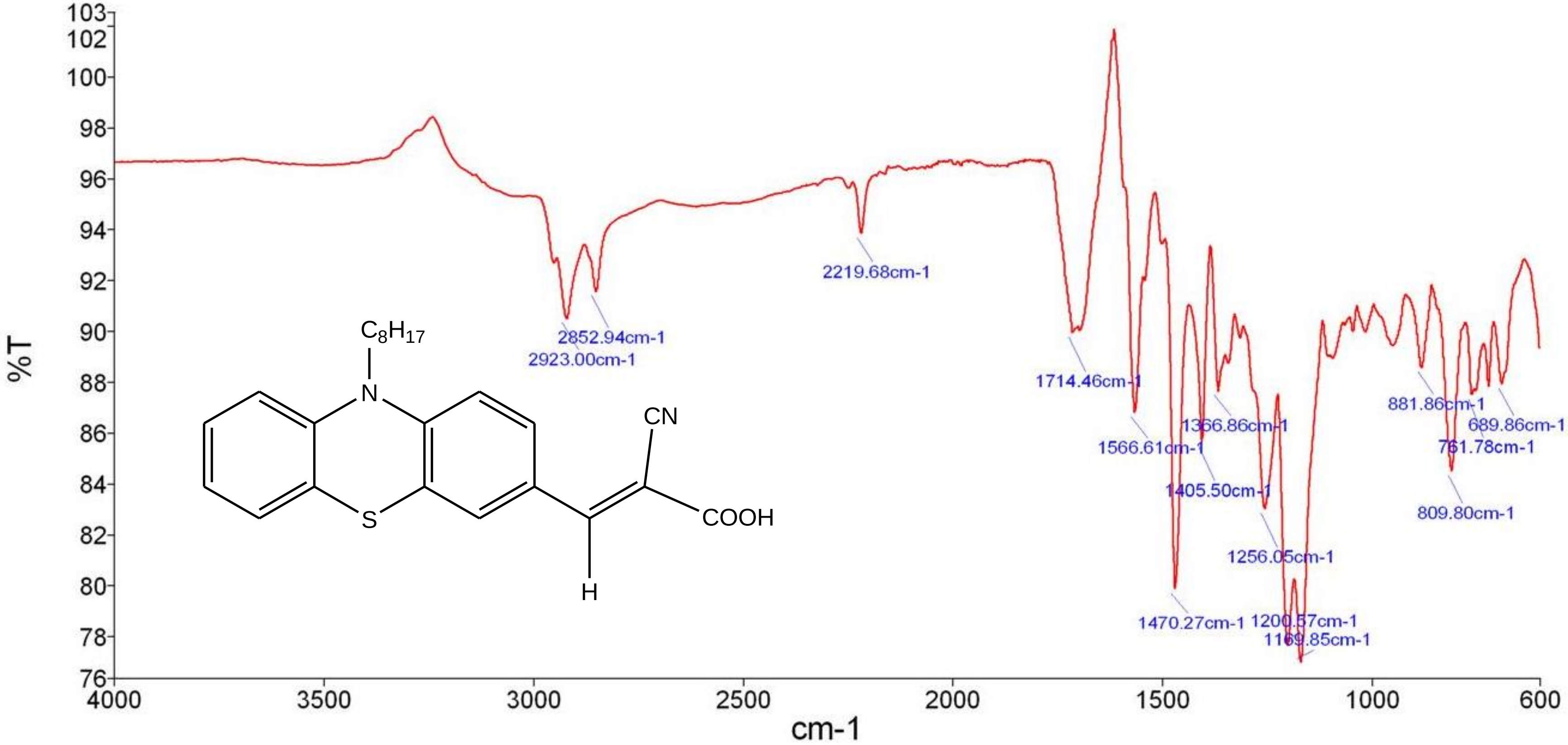

Figure S1. IR of (Z)-2-cyano-3-(10-octyl-4a,10a-dihydro-10H-phenothiazin-3-yl)acrylic acid

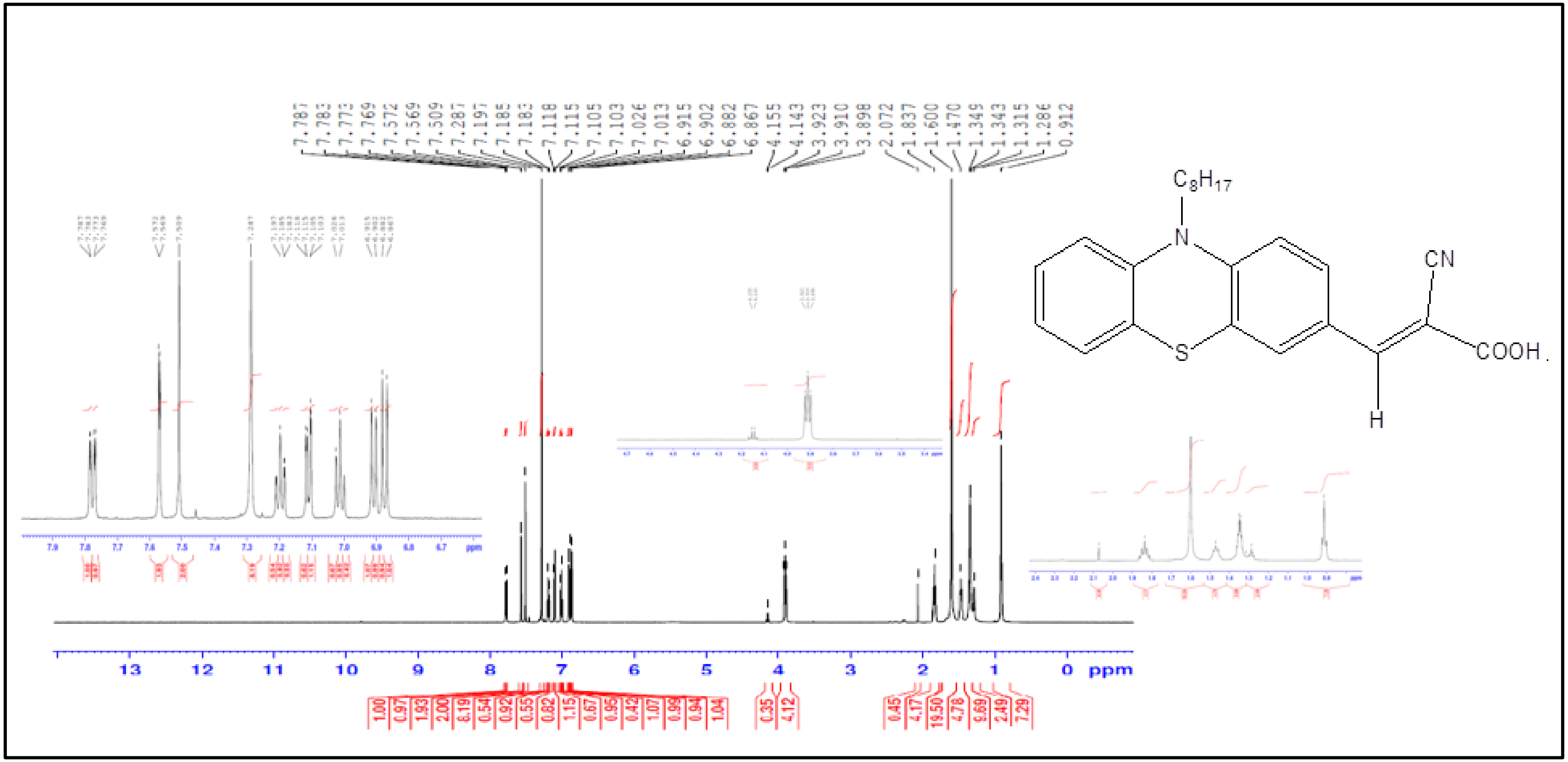

Figure S2. ^{1}H NMR of *(Z)-2-cyano-3-(10-octyl-4a,10a-dihydro-10H-phenothiazin-3-yl)acrylic acid*

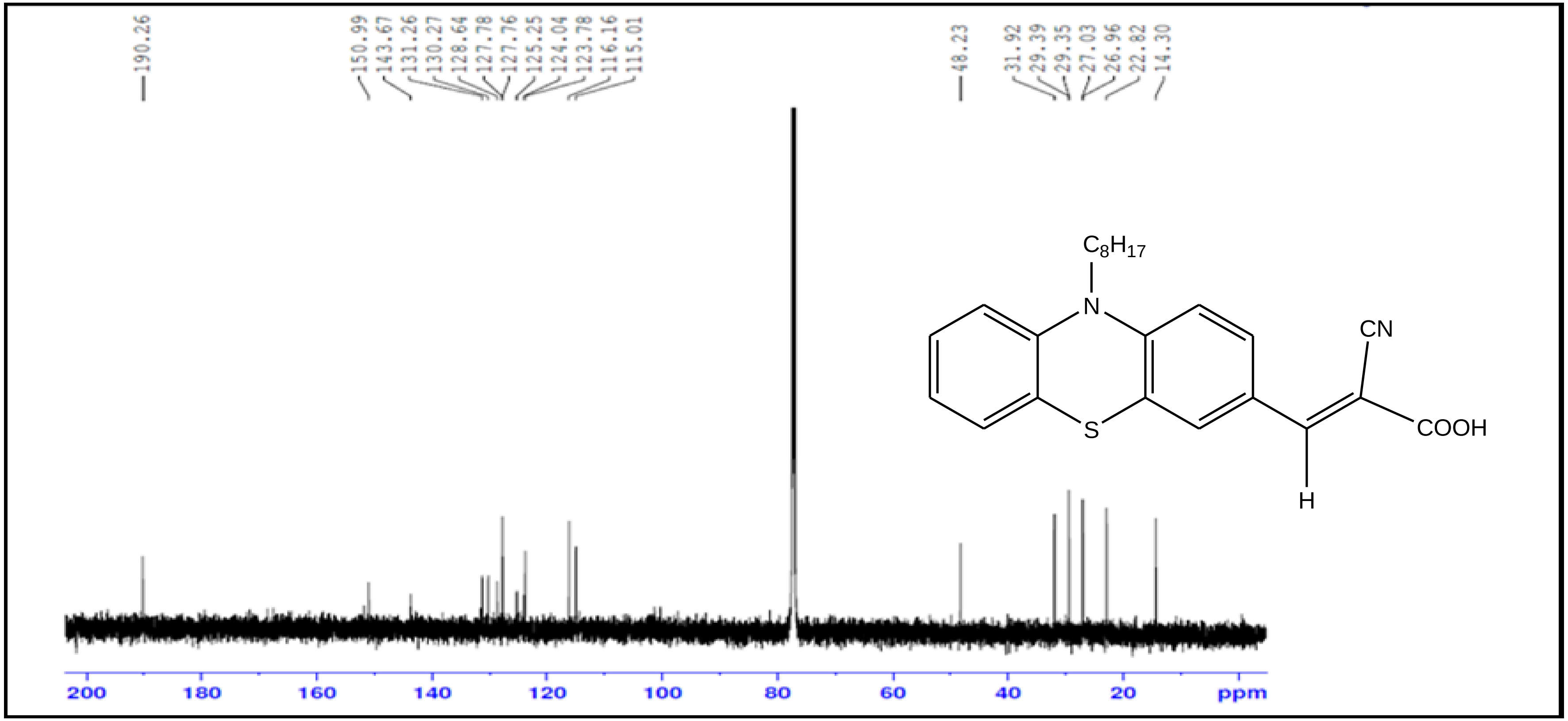

Figure S3.^{13}C NMR of *(Z)-2-cyano-3-(10-octyl-4a,10a-dihydro-10H-phenothiazin-3-yl)acrylic acid*

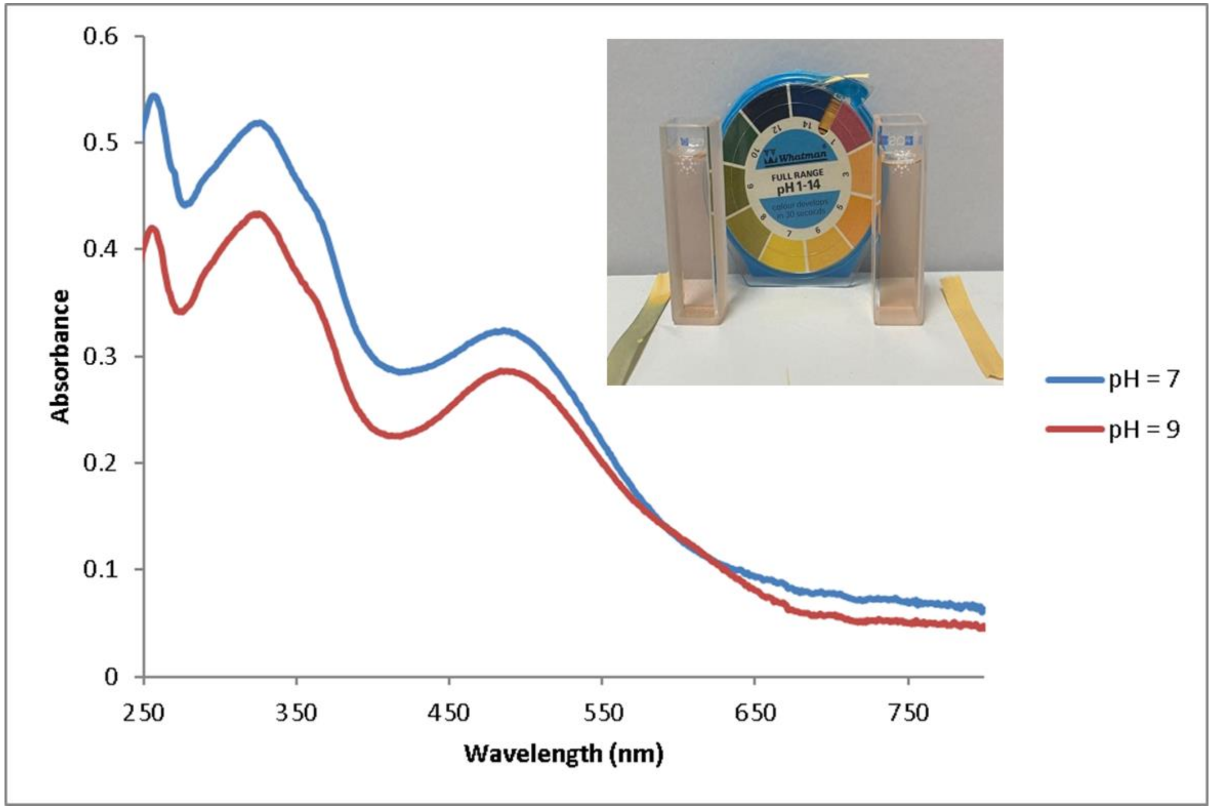

Figure S4. The stability of dye in basic medium at pH= 9.
